# Accidentally Swallowing a Toothbrush in a Patient during a Vomiting Attempt: Literature Review and Case Report

**DOI:** 10.3390/ijerph19052682

**Published:** 2022-02-25

**Authors:** Maria José Mesa-López, Carina Martínez-Camacho, Francisco Mesa

**Affiliations:** 1 Digestive Department, University Hospital Virgen de la Arrixaca, 30331 Murcia, Spain; mari_ml_2@hotmail.com (M.J.M.-L.); carina.z04@gmail.com (C.M.-C.); 2 Periodontology Department, School of Dentistry, University of Granada, 18071 Granada, Spain

**Keywords:** swallowing disorders, manual toothbrush, gastroscopy, case report

## Abstract

Swallowing a whole toothbrush is a rare event. As of today, no case described has documented that the foreign body has passed through the entire gastrointestinal tract and has been spontaneously eliminated. Places where it is most frequently retained have been described. Only in one single case described did the foreign body reach the colon. We describe the main injuries caused by this foreign body, and the most common correct therapeutic approach for solving the problem. The third case in the literature is presented, with diagnosis and treatment of a woman who, in an attempt to induce vomiting, swallowed a toothbrush which became lodged in her stomach. The patient, at the time of the examination, only showed abdominal pain and anxiety.

## 1. Introduction

The variety of non-food items that are placed in the mouth and then swallowed, either intentionally or by accident, is surprising. Swallowing a manual toothbrush, excluding broken toothbrush fragments, is a rare event [1]. The three most frequent causes are: accidentally after brushing the base of a coated tongue, psychological disorder (bulimia/anorexia nervosa, schizophrenia), and suicide. Literature was reviewed (MedLine, Scopus, Embase), using “swallowed and Toothbrushes” as search terms, with no time limit and in English. To our knowledge, there are no more than 50 cases described in 25 case reports.

There is no standard size of manual toothbrushes for adults. Their measurements are usually between 16–22 cm long (including head, neck and handle), of which 3 × 1.25 cm is the head (length × width) and 0.7–1.3 cm is handle’s width. Any entry into the gastrointestinal tract represents a particular problem, as it never passes through the gastrointestinal tract spontaneously. Normally, it is retained in the esophagus or reaches the stomach. Lee MR et al. describe the unique case of a 31-year-old man, diagnosed with schizophrenia, in which the toothbrush was found in the ascending colon and caused a fistula between the right colon and the liver. It had to be extracted via laparotomy [2]. Yaosaka T. et al. describe a case where the toothbrush was located in the second portion of the duodenum of a woman who swallowed it while trying to brush the base of her coated tongue [3]. Mokánszki I. and Adorján T. document the case of a 13-year-old girl who accidentally swallowed a toothbrush, the handle of which was retained in the pylorus and the head of which entered into the stomach [4].

The first option for a therapeutic approach, especially if a short time has passed since ingestion, is endoscopy. The first successful performance of this procedure was reported in 1983 [5]. When this technique fails to result in extraction, laparoscopy by gastrotomy is recommended as a second option [6,7].

The main complications caused by this foreign body (fb), excluding the lesions already mentioned, are pressure necrosis, gastritis, mucosal tears, perforation, subcutaneous emphysema, and bleeding [8]. An ulcer 1.5 cm from the pylorus was documented by Cox D. et al. in a 33-year-old woman who swallowed a toothbrush as a suicide attempt. Two years after that incident, she started to experience acute abdominal pain and was admitted to Emergency Services. Scans showed the fb in the stomach and a laparotomy resolved the issue [9].

Bulimia nervosa is a disabling psychiatric disorder characterized by recurrent binge-eating (consuming large amounts of food with a sense of lost control) and inappropriate compensatory behaviors (self-induced vomiting; laxative, diuretic, or medication misuse; and fasting or excessive exercise) aimed at preventing weight gain [10]. In all cases described in literature, only two cases correspond to two girls who accidentally swallowed their toothbrushes while inducing emesis. Both were diagnosed with bulimia nervosa [6]. Using the toothbrush as an instrument to stimulate vomiting, by rubbing the uvula or pharyngeal wall, is considered a common practice in people with this disorder. Accidentally swallowing the toothbrush may be a consequence of this obsessive behavior. This article describes the third case in the literature where a patient who, although not diagnosed with bulimia nervosa, admitted to frequently inducing vomiting.

## 2. Case Report

**Figure 1:**
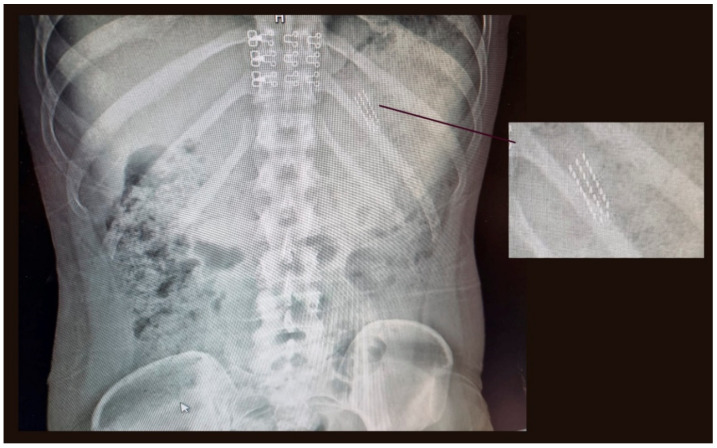
Chest and abdominal X-ray.

A 24-year-old woman was admitted to the Emergency Services of the Hospital Clínico Universitario Virgen de la Arrixaca (Murcia, Spain) in January 2022. She reported that she accidentally swallowed a manual toothbrush when inducing vomiting after a substantial Christmas meal. The patient immediately went to the hospital, there was a three-hour period between the time of the accident and the start of the examination, complaining of abdominal pain. She also suffered an anxiety attack at the time of the examination. Her other medical history is not relevant. Chest and abdominal X-rays were performed (Figure 1), showing toothbrush bristles in the gastric cavity, while the brush handle cannot be seen. Paracetamol—1 g IV, omeprazole—40 mg IV, and diazepam—2.5 mg IV were administered.

Upon observing the presence of the toothbrush in the gastric cavity, the anesthesiologist was contacted to perform an emergency gastroscopy with general anesthesia and orotracheal intubation to protect the airway. As the patient did not comply with the necessary fasting hours, the extraction was delayed for greater safety. Gastroscopy was finally performed in the operating room 6 h later, with extraction of the fb and satisfactory orotracheal extubation.

**Figure 2:**
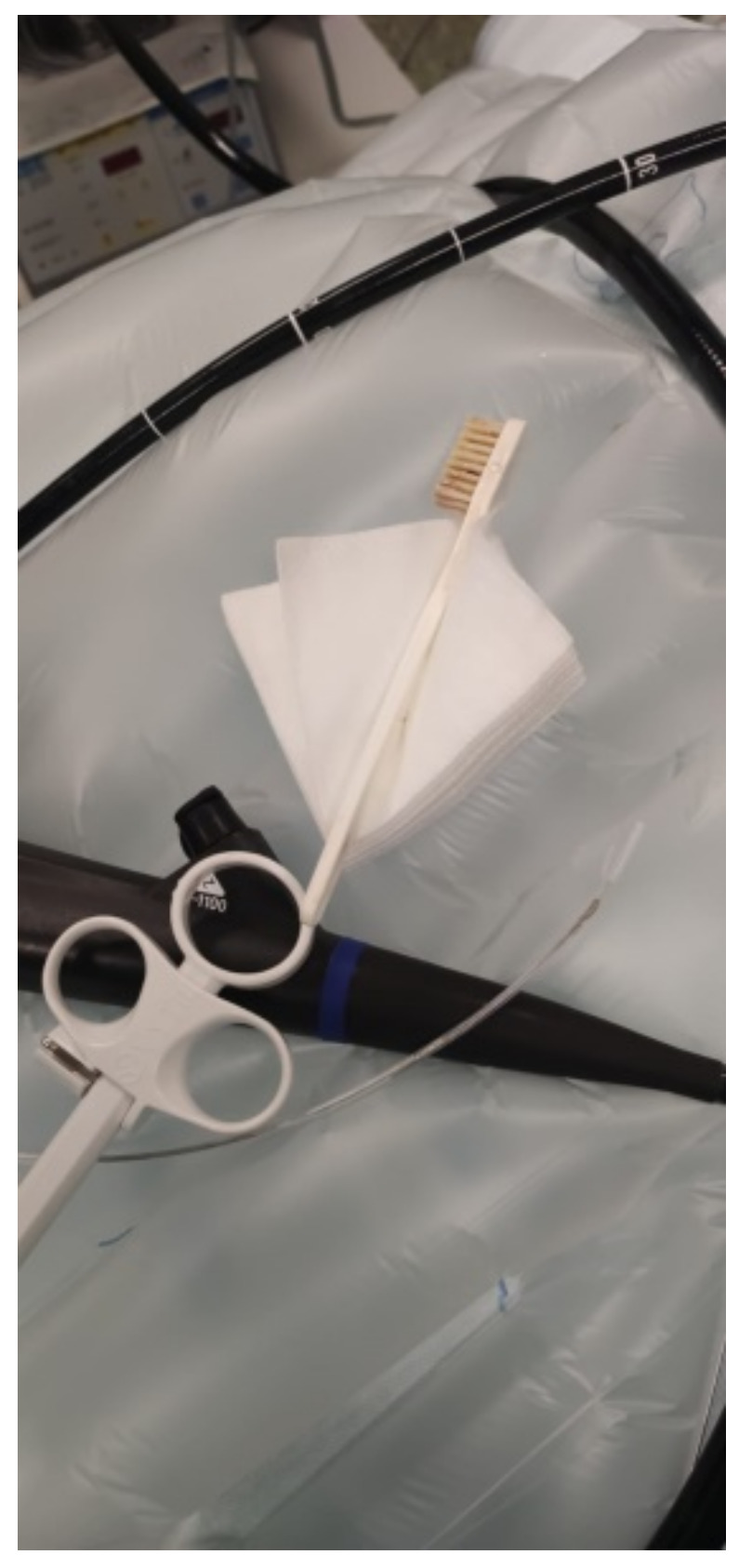
Image of the used endoscope and the toothbrush once removed.

The fb described above was observed in the stomach with no lesions on the oesophageal or gastric mucosa. The fb was successfully removed via endoscopy using a polypectomy snare videogastroscope Olympus GIF-1100 (Olympus Iberia, Barcelona, Spain) (Figure 2). After three hours of monitoring, the patient was discharged.

The patient also recognized that this procedure with a manual toothbrush was repeatedly conducted, especially in all those events where abundant meals took place. On the other hand, she did not refer to the use of laxatives or other types of medication to achieve this goal.

## 3. Discussion

In bulimia, the perception of the reality of the body itself is affected and patients force vomiting or take laxatives, to eliminate food intake as soon as possible. There are reported cases of approximately 40 episodes of induced vomiting per day, in one year [11]. It is estimated that over 50 million people in the world will develop this disorder at some point in their lives [12]. Using a toothbrush to make yourself vomit, and accidentally swallowing it, can be a clue for doctors and parents, in the diagnosis of this pathology [13]. In our case, the patient was not diagnosed with bulimia nervosa, nor were the parents aware of this practice to induce vomiting. However, the patient did acknowledge that after a copious Christmas meal, she forced herself to vomit with a manual toothbrush. In the radiological image, the characteristic image of the brush head can be seen, with three parallel rows of tufts of bristles, just at the level of the 11th left rib.

The majority of fb ingestions occur in the pediatric population. Most ingested fb pass spontaneously (80–90%). However, approximately 10–20% of cases of fb ingestion require endoscopic removal, while less than 1% will need surgery for foreign body extraction or to treat complications [14]. To date, no cases described in the literature on toothbrush swallowing, although for different reasons, documented the spontaneous elimination element. The esophagus is the most common anatomical site of large fb impactions, occurring in 75% of cases, due the cricopharyngeal sphincter. This sphincter is the narrowest point of the gastro-intestinal tract, since it measures approximately 14 mm in diameter [15]. A fb lodged in the stomach leads to few symptoms in the absence of complications. Urgent endoscopic removal (<12 h) of the fb with sharp morphology is recommended. Deferred endoscopy (<24 h) is used if fbs, greater than 5 cm in length or with a diameter greater than 2 cm, are blunt, since it is more feasible that they do not cross the pylorus [16]. Early diagnosis and treatment decrease morbidity and hospital stay. The use of antibiotics is controversial, as justified by Kim YH et al. to prevent complications [17]. In our case, it was not necessary because the exploration of the gastric mucosa did not show any complications.

Fbs that cross the pylorus are more likely to be located in the duodenal knee, at the level of the Treitz ligament or the ileocecal valve, and can cause occlusive symptoms. Those who fail to pass the distal duodenum within a week will be considered for surgical treatment [18]. In our case, due to the position of the head, it can be deduced that it is located vertically and in the upper half of the stomach, still separated from the pylorus, perhaps due to rapid medical intervention. For the endoscopic extraction of food boluses or fb, we have different endoscopic materials, such as polypectomy snare, Roth net, mouse or alligator tooth forceps, etc., depending on the morphology of the fb. The possibility of accidental introduction into the airway should be minimized (assessing orotracheal intubation) and damage to the esophageal or gastric mucosa should be avoided, using an overtube or a cap [18]. Therefore, caution is required as well as extensive experience of the endoscopist.

Some considerations for the extraction of this fb, considered large, according to Tonkic et al. [19], are the following. Firstly, the fb should aligned once it is caught in the stomach, with its longitudinal axis, parallel to the esophagus. This is especially important when pulling the object through the cardias. In this sense, there are authors who affirm that when the toothbrush is lodged horizontally in the gastric body and due to the shape of the stomach, endoscopic extraction is difficult [7]. Otherwise, this most critical and demanding part of the extraction procedure may easily result in mucosal damage (laceration and bleeding) or fb impaction. The second step in the extraction procedure is when the fb reaches the oropharynx. The patient has to extend his head backwards and the endoscopist has to reach for the toothbrush with his hand and pull it out [19].

In the case we describe, the patient was intubated orotracheally and a polypectomy snare was used with the endoscope. Lastly, the need for an endoscopist operator in the emergency room should be noted as a drawback, as some experience and familiarity with the equipment and materials are necessary to extract the material.

## 4. Conclusions

A rare event of this type occurred in a young woman with an eating disorder, which should be properly diagnosed. A rapid diagnosis, followed up by treatment, is imperative. The location and state of a fb must be determined through a careful imaging analysis. An early endoscopic procedure with the appropriate endoscopic equipment and operator experience will satisfactorily resolve the emergency.

## Data Availability

Not applicable.
